# Conference Report: LPMHealthcare Emerging Viruses 2023 (EVOX23): Pandemics—Learning from the Past and Present to Prepare for the Future

**DOI:** 10.3390/pathogens13080679

**Published:** 2024-08-10

**Authors:** Fern Jenkins, Tobias Mapulanga, Gauri Thapa, Kelly A. S. da Costa, Nigel J. Temperton

**Affiliations:** 1 UKHSA, Porton Down, Salisbury SP4 0JG, UK; fern.jenkins@ukhsa.gov.uk; 2 Medway School of Pharmacy, The Universities of Kent and Greenwich at Medway, Chatham ME4 4BF, UK; tmm44@kent.ac.uk (T.M.); gt301@kent.ac.uk (G.T.)

**Keywords:** pandemics, preparedness, zoonotic viruses, methodology development

## Abstract

The emergence of SARS-CoV-2 has meant that pandemic preparedness has become a major focus of the global scientific community. Gathered in the historic St Edmund Hall college in Oxford, the one-day LPMHealthcare conference on emerging viruses (6 September 2023) sought to review and learn from past pandemics—the current SARS-CoV-2 pandemic and the Mpox outbreak—and then look towards potential future pandemics. This includes an emphasis on monitoring the “traditional” reservoirs of viruses with zoonotic potential, as well as possible new sources of spillover events, e.g., bats, which we are coming into closer contact with due to climate change and the impacts of human activities on habitats. Continued vigilance and investment into creative scientific solutions is required for issues including the long-term physical and psychological effects of COVID-19, i.e., long COVID. The evaluation of current systems, including environmental monitoring, communication (with the public, regulatory authorities, and governments), and training; assessment of the effectiveness of the technologies/assays we have in place currently; and lobbying of the government and the public to work with scientists are all required in order to build trust moving forward. Overall, the SARS-CoV-2 pandemic has shown how many sectors can work together to achieve a global impact in times of crisis.

## 1. Introduction

**Figure 1:**
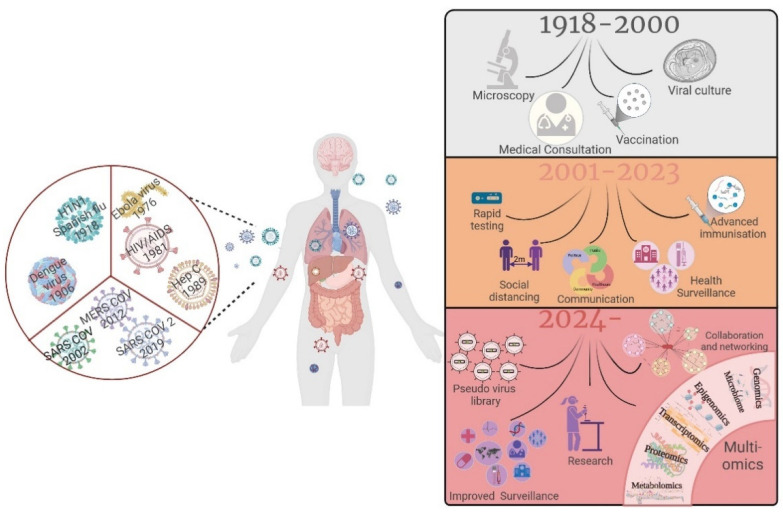
Schematic showing latest emerging virus R&D which builds successively on previous epidemics and pandemics. Figure generated using BioRender.

The emergence of SARS-CoV-2 has brought pandemic preparedness onto the global scientific radar. Taking place in the historic “Teddy Hall” location in Oxford, the one-day LPMHealthcare conference on emerging viruses (6 September 2023) was attended by delegates from academia, public and animal health institutes, and representatives from industries that participated in the efforts to combat the SARS-CoV-2 pandemic, with the aim to prepare for ‘disease X’ and the next pandemic. The speakers were experts in vaccine and therapeutics discovery, methodology and technological development, and surveillance. Presentations highlighted research on all aspects of pandemic preparedness, from monitoring both “traditional” reservoirs of viruses with zoonotic potential to possible new sources of spillover events from species such as bats, which we are being brought closer to due to climate change and the impacts of human activities on habitats. Additionally, the meeting showcased novel methods of surveillance and vaccine testing, as well as innovative drug discovery approaches over the last few years. The idea behind the conference was that collectively reviewing efforts made during the pandemic would lead to discussions about best practice; new approaches to embrace in preparation for the future; and potential collaborative networks that will be ready for the next pandemic (summarized in Figure 1).

## 2. Pandemics Past

**Professor John Oxford** (Queen Mary University of London) opened proceedings by providing an in-depth look at the first fully documented global influenza H1N1 pandemic in 1918 (referred to as the Spanish flu) [1,2]. The migration patterns of birds played a key role in antigenic shift changes due to avian species mixing along these pathways, illustrating the importance of monitoring wild and domestic bird populations. A spillover event clearly occurred from birds to mammals, then to humans, and, disturbingly, it proved fatal for a large number of young people; the spread was clearly linked to troop movements during World War I [3]. Oxford used a variety of photos to bring home the human cost of the 1918 ‘Spanish flu’ while demonstrating the effect human behaviour can have on the impact of viral spread. For example, the use of face masks or cloths as shields, the rules banning gatherings, the closing of schools, and other social distancing practices used in 1918 have been assessed as successful in lowering mortality rates [4]. The successes and failures of the preventative measures used then, and how these informed the measures taken to combat SARS-CoV-2, was discussed. Additionally, the study of carefully collected and stored samples from patients infected with the 1918 ‘Spanish flu’ can be used to infer future risks from influenza and provide insight into viral evolution and pandemic potential [5]. Indeed, the haemagglutinin (HA) from the 1918 H1N1 virus and the most recent pandemic H1N1 influenza strain (2009) are almost completely homologous [6].

## 3. Pandemics Present

The ‘Pandemics Present’ section of the conference began with a keynote talk from **Professor Danny Altmann** (Imperial College London, UK) titled ‘Immune phenotypes in acute COVID-19 and Long COVID-19.’ Although it has only been a few years since the start of the COVID-19 pandemic, the population has developed a heterogenous hybrid immunity due to a diverse range of infections coupled with a diverse vaccine history. Whilst many lessons have been learnt on the human immunology of COVID-19, researchers are left with the puzzling challenge of prolonged symptoms long after the acute infection subsides: long COVID. The ever-evolving symptom list ranges significantly from mild to profoundly debilitating, spans multiple organs and systems, and occurs regardless of variant. Yet, the biological underpinnings are poorly understood. Currently, very few studies have been conducted with a focus on selecting patients based on their symptoms and affected organs, and, thus, their disease mechanisms. Without this, there is a risk that clinical trials may falsely show that a treatment has no benefit if patients with a multitude of pathogenic pathways are included in the same study. It is hypothesised that different clusters of symptoms are driven by distinct mechanisms. Felicity Liew [7] determined that patients can be stratified based on their symptom clusters, which correlate with differential serum markers. Further to this, Maxime Taquet [8] identified two distinct biomarker profiles measured during acute admission which could predict cognitive defects at 6 and 12 months post-infection. This study adds evidence that long COVID symptoms arise from different causes, and that therapeutic trials need to take this diversity into account. Altmann then moved on to his own key paper: ‘The Immunology of Long COVID’ [9], which attempts to link the proposed consequences of COVID to their potential contribution to long COVID. The hypothetical mechanisms include ideas such as a persistent SARS-CoV-2 reservoir, reactivation of the Epstein–Barr virus, or autoimmunity. For example, persistent virus or antigen reservoirs due to COVID-19 may cause ongoing immune activation, therefore contributing to long COVID. With each new variant of concern (VOC), the burden of long COVID cases is predicted to cumulatively rise. The major challenge is how to stratify patients into clusters. Those affected are waiting for research to be translated into clinical trials and treatments, and we will likely see the best results when the diagnosis and phenotypes are better clarified. Thus, the current picture is one of convergence towards a map of the immunopathogenic aetiology of long COVID, though the data are as yet insufficient to perform mechanistic synthesis or to fully inform therapeutic pathways.

Several speakers presented talks on current approaches to monitor and study SARS-CoV-2, starting with **Professor Nigel Temperton** (Viral Pseudotype Unit, University of Kent), who presented his work ‘Pseudotyping the Pandemic’. At the beginning of the pandemic, the Temperton laboratory quickly developed lentiviral-based SARS-CoV-2 pseudotyped virus (PVs) [10] and became involved in one of the first pseudotype neutralisation (pMN) studies to estimate the seroprevalence of SARS-CoV-2 in March–May 2020 [11], which offered an insight into asymptomatic infection and pre-existing immunity. SARS-CoV-2 variants of concern (VOC) and the continual rapid evolution of the virus remain a challenge as mutations impact infectivity and immune evasion (natural or vaccine-generated responses). The pseudotyping system allows one to quickly update the platform according to the circulating VOCs. Temperton *et al*. utilised PVs from Wuhan expressing spike proteins, as well as circulating VOCs, to highlight a significant reduction in the ability of first wave convalescent sera to neutralise the VOCs P.1 and B.1.351. This paper demonstrates the usefulness of pseudotyping systems to quickly assess the ever-evolving variants that will have to be monitored for the foreseeable future [12]. A further study examined reinfection cases with individuals infected one time pre-vaccination [13]. This study utilised anti-SARS-CoV-2 nucleocapsid and both live virus microneutralization (LV-MN) and pMN assays, and was able to determine titres associated with significant reductions in the odds of reinfection. For pMN this represented a titre above 100, and for LV-MN, titres above 40 against the Wuhan strain [13]. Whilst PVs have been used as a surrogate to safely study high-consequence viruses, many question how strongly the results correlate with traditional assays, e.g., LV-MN. The VPU carried out a systematic review that clearly shows that SARS-CoV-2 pMN correlates strongly to LV-MN across many individual studies [14]. However, it was noted that care must be taken when considering the core and reporter to be used when generating new pseudotyping systems, as these can affect reproducibility [14]. Building on this, Temperton theorised that PV libraries could be used to screen sera to determine how well-protected we are from other viruses that may “jump” into humans. For example, pMN assay was used to demonstrate the ability of convalescent and vaccination sera to successfully neutralise the bat coronavirus RaTG13 [15]. This and other studies clearly demonstrate that the pMN system can also be used to generate new mutations in the spike proteins to predict the protective effects of vaccination or natural immunity as circulating strains of SARS-CoV-2 change [16,17,18,19].

**Dr Giada Mattiuzzo** (Medicines and Healthcare Products Regulatory Agency, UK) discussed ‘The role and importance of reference materials for emerging virus outbreaks’. Standards are of key importance for the manufacture of highly complex biological products, which must be measured for their biological effect in an appropriate unitage. WHO International Standards (IS) are established by the World Health Organisation (WHO)’s Expert Committee on Biological Standardisation and are the highest order of reference material for the calibration of assays. IS are calibrated in units of biological activity, which are assigned following extensive studies across multiple laboratories, and allow for harmonisation and, thus, comparability of the results of assays measuring biological activity. WHO IS are assigned potencies in International Units (IU) which quantify the amount of biological activity present in a sample. Mattiuzzo used the example of Lassa virus (LASV), an endemic virus in several West African countries that is classed as a top 10 priority pathogen for its outbreak potential and lack of effective treatment, to demonstrate the role of IS in analysing vaccine efficacy and the reliability of assays to measure the antibody response, thereby allowing accurate comparisons between laboratories. The candidate preparation sample was assessed in a sample panel which included the WHO international reference panel. The candidate IS was detected in all neutralising assays and binding assays for IgG and so was assigned as the first WHO IS for anti-Lassa virus IgG, with an arbitrary unitage of 25 IU/ampoule for neutralising activity. The IS for SARS-CoV-2 immunoglobulin was generated following an international collaboration between laboratories from 15 countries, using a wide range of methods [20]. However, there are a range of issues preventing the best use of IS. Firstly, researchers may misunderstand the official role of the IS and misuse it, such as using the IS as a positive control or as a validation tool which causes stock to run out quickly. Moreover, researchers may not know how to effectively calibrate an IS, which Mattiuzzo plans to change by using webinars and advertising the WHO manual. The biggest issue related to IS usage is that production time does not correspond to immediate needs. WHO international standards take 2–3 years to produce, and in this time, researchers will have generated assays with their own unitage. During the outbreak of the new high-consequence virus SARS-CoV-2, the team utilised high-titre convalescent plasma from one recovered patient as a reference reagent for the anti-SARS-CoV-2 antibody. Researchers would use this reagent to standardise serological assays and compare datasets across laboratories to determine the antibody levels needed in vaccines. Once a SARS-CoV-2 IS was developed, it was retrospectively calibrated, and data could be converted into international units. Overall, many standards for known high-consequence viral pathogens are available, or are in production in case of the need to rapidly respond to potential outbreaks. Research reagents can be produced quickly to provide a common material as a substitute until a WHO IS can be generated.

In contrast, **Dr Lucy Thorne** (University College London) looked at the role of innate immunity in her presentation ‘The convergent evolution of SARS-CoV-2 variants to enhance innate immune suppression.’ The innate immune system is a universal barrier to infection—all viruses must evade it to emerge and transmit. However, the innate system is also a central regulator of emerging virus transmission, pandemic potential, and pathogenesis. Therefore, the key question asked by Dr Thorne is as follows: how did SARS-CoV-2 overcome innate immunity? There is evidence that SARS-CoV-2 emerged with the ability to manipulate human innate immunity, by activating a delayed innate immune response [21]. Dr Thorne found that SARS-CoV-2 replicates rapidly in Calu-3 lung epithelial cells and, despite triggering an immune response through the activation of RIG-I and MDA5 (cytoplasmic RNA sensors) and inducing cytokines, chemokines, and interferons, as well as an IFN-stimulated gene expression signature (ISG), the IFN response appears too late to suppress SARS-CoV-2 replication. The emergence of its VOCs suggests viral adaptation to enhance transmission. Isolates of the Alpha variant (B.1.1.7) were found to suppress innate immune responses in airway epithelial cells more efficiently than first wave isolates [22]. The Alpha variant upregulated three key innate immune antagonists: nucleocapsid (N), Orf9b, and Orf6, which are hypothesised to enhance replication and delay early host innate immune responses, potentially through a similar mechanism to HIV innate immune evasion [23]. In addition to mutations in spike (S) proteins, VOCs gain mutations in non-structural, structural, and accessory proteins (e.g., Orf9b, Orf6, and N) [24], as reported in the N and Orf9b regulatory regions of the Delta and Omicron variants [22]. When comparing the VOCs to the Wuhan strain, it was determined that variants Alpha through to Delta all convergently evolved enhanced ISG suppression. Omicron BA.1 is the exception. ISG suppression correlates with the expression of viral innate immune antagonist proteins including Orf6, N, and Orf9b [25], suggesting that innate immunity is an important selection pressure for virus evolution. Identification of the molecular players underlying process this may lay the groundwork for how we examine VOCs, tackle future pandemics, and develop novel treatments.

**Dr Ashley Otter** (UK Health Security Agency) leads the emerging pathogen serology group that was born out of the COVID-19 pandemic. The group is involved in sero-surveillance, assay development, and service testing for a wide range of pathogens. The group is also preparing for ‘disease X’ to best support UKHSA in the case of a new pandemic. Their main capability is high-throughput serology for emerging pathogens, to determine their immunology and prevalence in the population, with the capability to scale up processing to cover ~4000 samples per day.

Otter’s talk focused on the group’s recent involvement with the monkeypox (Mpox) outbreak, which saw 85,000 global cases and 3500 cases in the UK. The smallpox vaccine, IMVANEX (UK) or ACAM2000 (US), was offered to those at high risk to protect and limit spread. However, the effectiveness of these vaccines and any differences in protective mechanisms were unknown, and there were no commercial kits available to detect either the antibody response to vaccinations or infected individuals, and no way to distinguish between those exhibiting an antibody response and those infected. To resolve these issues, the team used principal component analysis (PCA) to identify antigens that demonstrate distinct binding variables between the two vaccinated groups, Mpox-infected patients and negatives. In total, 27 commercial poxvirus antigens (24 monkeypox virus and 3 vaccinia virus) were tested using ELISAs against sera from patients with different doses of the IMVANEX vaccine and convalescent sera from naturally infected individuals to study poxvirus-induced antibodies. Using this approach, it was found that the negative sera group and individuals who had received one dose of IMVANEX were not distinguishable from each other. Those with two doses of IMVANEX showed separate clusters distinct from the negative and one-dose groups, and positively correlated with those who had received the ACAM2000 vaccine, as well as convalescent Mpox sera. Mpox convalescent sera were highly correlated to each other, but also with smallpox-vaccinated individuals. Infection with Mpox leads to an antibody response to a range of poxvirus antigens that is similar to that seen in smallpox-vaccinated patients. The MPXV antigen A27 response was identified in Mpox-infected patients and ACAM2000-vaccinated patients, with the caveat that as A27 is used in ACAM2000 (found in the US only), this antigen can only be used when investigating UK-based individuals. Therefore, it is possible to distinguish Mpox-vaccinated from Mpox-infected patients in the UK as long as they have not received the smallpox vaccine [26]. This approach of high-throughput epitope mapping and scaling up binding and neutralisation assays provides the framework for designing novel vaccines and rapidly assessing vaccine efficacy.

## 4. Pandemics Future

**Professor Ian Brown**, (Animal and Plant Health Agency (APHA)) opened the Pandemics Future section of the conference with his keynote speech looking at ‘Avian Influenza: the next pandemic?’ Avian influenza is a high priority on the UK government risk register. There is concern about an observed increase in the highly pathogenic avian influenza (HPAI) virus in birds globally. Of particular concern are the emergence, fast evolution, and rapid spread of the influenza A virus (IAV) H5 subtype HPAI viruses, known as clade 2.3.4.4b, among birds across various continents, coupled with sporadic reports of human transmission [27]. The good news is that despite the substantial interface between humans and domesticated birds there have been relatively few reports of infection in humans, and to date, there have not been any reports of human–human transmission. Additionally, there is no evidence that human-adapted influenza viruses, e.g., H1N1 from 1918, have undergone reassortment with human influenza viruses [28]. This does not preclude the possibility of a zoonotic spillover event resulting in pandemic influenza in the human population, especially when mutations in viral polymerase and the acquisition of neuraminidase (NA) from coinfected birds are possible, both of which could potentially enhance the virus’s ability to cross species barriers, infecting a broader range of wildlife populations, particularly mammals. Professor Brown gave the example of foxes that had contracted influenza after eating dead birds [29,30]. The environment is hard to control, especially when dealing with wildlife. Instead, Professor Brown advocated for increased international surveillance of wild and domesticated birds, combined with continued research into circulating IAV strains, with a One Health approach connecting research into animal and human strains of IAV, and he also advocated for the consideration of vaccination programmes.

With increased attention focused on coronaviruses since the SARS-CoV-2 pandemic, **Dr. Rachel Tarlinton** (University of Nottingham) highlighted the diversity of coronaviruses and their ability to infect a wide range of species, leading to respiratory or gastrointestinal diseases. Many mammals, including humans, have multiple coronaviruses from the alpha and beta genera. SARS-CoV-2 in particular is able to infect a wide range of species, from rodents and mustelids to felines and primates. Interestingly, although dogs have been found to be infected with SARS-CoV-2, the virus does not replicate well and therefore does not appear to transmit to others, whereas there has been evidence of human-to-animal and then animal-to-human transmission in cats, farmed mink, and deer [31,32,33]. Since the SARS-CoV-2 pandemic, more studies are investigating Sarbecoviruses in bats, identifying factors which may confer potential to transmit to humans [34]. In the context of European wildlife studies, Tarlinton discussed the fact there is a general scarcity of widespread SARS-CoV-2 infection, with occasional cases reported in mustelids and deer. Ongoing, species-specific sampling efforts are yielding diverse results [35,36]; of note, some of these viruses have been found in human pneumonia cases, raising concerns about potential public health implications [37]. Various animal models have been used in the laboratory to study SARS-CoV-2 transmission, such as ferrets, non-human primates, rats, mice, and hamsters [38]. Overall, the dynamic interactions of coronaviruses with diverse animal species affect the susceptibility of different animals to SARS-CoV-2 and the potential for cross-species transmission. The complex nature of these viruses in both natural and laboratory settings underscores the importance of ongoing studies and monitoring efforts for a better understanding of the disease’s ecology and potential public health implications. This will require continued support and funding from the public and governments.

**Dr. Gábor Kemenesi** (University of Pecs, Hungary) presented work on the recently described Lloviu virus, discovered in Spain in 2011 in Schreiber’s bat [39]. This is a negative strand, non-segmented RNA filovirus belonging to the genus Cuevavirus, and is related to the Ebola and Marburg viruses [39]. Since then, the virus has been identified in Hungary, Italy, Bulgaria, Serbia, Slovenia, and Romania, all within the same bat species [40,41,42]. Detection of the virus is feasible in various bodily fluids, including blood, urine, guano, and saliva, as well as in infected organs and ectoparasites of the affected bats. Kemenesi and his research team employed various methods, including GP-based pMN, qRT-PCR, viral genome sequencing, and in vitro virus assays, to characterise the Lloviu virus. The generation of novel genomic data from 32 genomes enabled the analysis of evolutionary and genomic characteristics. Notably, identical Lloviu virus genome sequences were identified in both bats and parasites. These findings corroborate earlier research and affirm that the glycoprotein (GP2) gene in the Lloviu genome is a hotspot for mutations [40]. The prevalence of Lloviu virus was reported as 1.14% in live animals and 18% in ectoparasites. The researchers have proposed the likelihood of multiple divergent LLOV strains from various lineages circulating across the entire geographic range of Schreiber’s bat. More importantly, there is currently no evidence of bat-to-human transmission, especially considering the limited contact between bats and humans [41]. The researchers did not detect any functional adaptation in the Lloviu virus sequence that developed after 2002 [43]. The studied bats exhibited higher levels of neutralizing antibodies at the end of the hibernation period compared to live animals sampled during the summer and autumn. Two juveniles, aged between 1.5 and 2 months, were found to have likely acquired maternal immunity to Lloviu virus. PCR testing of the two juveniles was negative for LLOV, and they exhibited low levels of LLOV-neutralizing antibodies [43]. Interestingly, sick bats were observed hibernating separately from the main colony, suggesting a possible behavioural response to illness [44]. Overall, there is no definitive indication that this virus does represent a threat to human health; however, as we come into contact with bats more often following climate change and loss of their habitat, it is important to characterise these viruses and assess their prevalence now.

With regard to ‘disease X’ causing the next pandemic, three of the speakers expanded on the use of pseudotyped viruses (PVs) in new vaccine and treatment evaluation, as well as the discovery of novel subtypes of viruses. **Dr Kelly da Costa** (Viral Pseudotype Unit, University of Kent) showcased the existing and expanding influenza pseudotype libraires available at the VPU. A growing collection of viruses express the HA glycoprotein of IAV (subtypes H1-H18), and the NA glycoprotein (N1-N11), influenza B (IBV) (HA and NA), and haemagglutinin-esterase-fusion (HEF) from influenzas C and D (ICV and IDV) [45,46]. The major advantage of using PVs is that they are safe and flexible tools which can be used to assess antibody responses to novel influenza strains. da Costa demonstrated their use in pMN for neutralising antibody assessments of polyclonal (serum) and monoclonal antibodies [45,47,48,49], as well as in pseudotype Enzyme-Linked Lectin Assay (pELLA) to assess the inhibition of NA activity [46]. This wide range of IAV, IBV, ICV, and IDV viruses encompasses a system that applies to all influenza viruses, including those with zoonotic potential, and embraces the One Health approach.

**Dr. Edward Wright** (Viral Pseudotype Unit, University of Sussex) focused on the significant impact of lyssaviruses as zoonotic agents. The lyssavirus genus, consisting of 20 species, is categorized into phylogroups (PG) I, II, and III based on genetic and antigenic diversity [50]. The rabies virus (RABV) is the causative agent of rabies encephalitis, which results in over 59,000 human deaths annually. Humans are usually infected after receiving a bite from an infected dog, presenting a substantial risk to 50% of the human population in Africa and Asia [51]. There is a lack of effective treatments for rabies symptoms, with preventive measures relying on vaccination. These vaccines offer varying levels of protection against phylogroup I (PG I) lyssaviruses, but do not extend their protection to viruses in phylogroups II and III. This limitation is mainly due to vaccines being based on inactivated RABV. Consequently, there is a crucial need for research to assess vaccine efficacy against newly identified lyssavirus species and develop novel vaccine antigens capable of eliciting robust, broadly neutralizing protection against all lyssavirus species [50,52]. Antibodies stimulated by RABV vaccines do not neutralize phylogroup II and III lyssaviruses. Wright spoke of an extensive, long-term study which is currently underway to characterize epitopes within the glycoproteins of all lyssavirus species. The information obtained, combined with a bioinformatic approach, will be utilised with the aim of developing antigens capable of providing broad protection against all lyssaviruses. Dr. Wright highlighted the role of PVs in developing novel vaccines in a study utilizing a reverse genetics system and a unique set of lyssavirus sera, where researchers characterized a recombinant virus, as well as PGI pseudotyped viruses, specifically the Taiwan bat lyssavirus (TWBLV) and the Kotalahti bat lyssavirus (KHUV). The findings revealed cross-reactivity with antibodies induced by RABV vaccines, although higher antibody levels were required to inhibit infection compared to the 0.5 IU/mL threshold for blocking RABV [53].

**Dr Simon Scott** (Viral Pseudotype Unit, University of Kent) described attempts to characterise a novel influenza virus isolated from a cloacal swab sample taken from a Common Pochard (Aythya ferina) in Kazakhstan in 2008. The partial viral sequence obtained in the field appears to have an HA which is not able to be characterised into any of the IAV subtypes H1-H18 [54]. This novel virus, referred to as Kz52 HA, has a coding sequence with only 68.2% nucleotide and 68.5% amino acid homology, with its closest relative in the H9 (N2) subtype [55]. Scott reported that their efforts to generate pseudotyped viruses (PVs) expressing Kz52 HA using various techniques have proved unsuccessful. These techniques included lentivirus and rhabdovirus cores; native or human codon-optimized HA ORFs; diverse plasmid expression vectors; different target cell lines; and a range of candidate proteases for HA cleavage. None of these approaches yielded Kz52 HA-pseudotyped particle supernatants with significant titres, unlike the successful H9 control, even after converting the cleavage site into a polybasic one. Immunofluorescence analysis of HA expression on the surface of HEK293 producer cells using an H9-specific primary antibody indicated H9 expression and, to a lesser extent, the presence of Kz52 HA. Additionally, a lower, yet still significant ELISA signal was observed for the Kz52 PV supernatant concentrates compared to H9. Dr Scott noted that this observation could suggest detection via H9 antibody cross-reactivity or simply lower expression levels, potentially affecting high-titre PV production. Research to find an identical isolate in the field continues alongside this work.

Finally, **Dr Laura Martin-Sancho** (Imperial College, London) presented a different approach to elucidating dengue immunology in her presentation “Systems biology approaches reveal host defence mechanisms against Dengue virus”. Dengue virus (DNV) is the most prevalent mosquito-transmitted virus, and its prevalence has been on the rise since the 1950s. The spread of DNV is accelerated by urbanisation and, importantly, climate change, with more than 300 million cases annually; currently there is a limited uptake of vaccines and a lack of effective antiviral treatments. To rectify this, an improved understanding of DNV itself and the frontline host defences that control DNV, including its internal spread and susceptibility to antibody-dependent enhancement (ADE), is required [56]. It is understood from other pathogens that innate defences determine the outcome of the infection, the pathogenesis, and the host range; influenza A virus is restricted by interferon-inducible transmembrane (IFITM) proteins [57], and 2′-5′-oligoadenylate synthetase 1 (OAS1) binds to dsRNA structures in the SARS-CoV-2 5′-UTR and blocks SARS-CoV-2 replication [58]. Dr Martin-Sancho aims to define the genes and networks that restrict DNV infection at a global scale. This is crucial in furthering our understanding of disease susceptibility and the knowledge will inform therapeutic design.

Dr Martin-Sancho’s laboratory uses a systems approach that yields multi-OMIC data to enable global dissection-based approach antiviral defences against DNV. In systems biology, high-throughput ‘omic’ techniques are employed to measure each element within the system. These elements may include DNA sequences, RNA and protein quantifications, interactions between proteins, protein and DNA, biological modules, signalling pathways, gene regulatory networks, cells, organs, individuals, populations, and ecosystems [59]. Theoretical examination spans from the cellular level to the entire organism, reflecting a holistic approach rather than a reductionist one [60]. Functional genomics data are necessary for the identification of antiviral host factors. These are obtained through transcriptomics, which brings to light host factors regulated by DNV infection, and proteomics, which yields data on protein abundance and DNV–host protein-to-protein interaction (PPI). Dr Martin-Sancho explained that previous research revealed the landscape of IAV restriction and that the integration of OMICs data from functional genomics, transcriptomics, and proteomics, followed by data modelling, helped to define the landscape of intrinsic antiviral defences against IAV. Using a large-scale gain-of-function analysis to evaluate the impact of human ISGs on viral replication, [61] previously identified broad-acting antiviral ISGs and eight ISGs that specifically inhibit SARS-CoV-2 and SARS-CoV-1. Among the broad-acting ISGs is BST2/tetherin, which inhibits viral egress and is antagonized by SARS-CoV-2 Orf7a protein [62]. To enhance our understanding of DNV biology and the influence of host factors in restricting infection spread, Dr Martin-Sancho and her colleagues adopted a systems biology approach when investigating the interplay between various host and viral factors in dengue infection in vitro, utilizing disease-relevant monocyte-derived dendritic cells (MDDCs) infected with a clinical isolate of DNV sourced from Nicaragua. Using cutting-edge technologies, the researchers conducted a comprehensive genome-wide genetic screen to pinpoint host factors that impede DNV, and subsequently analysed the effects of DNV infection on both the transcriptome and the proteome. They integrated these three datasets with previously published DNV–host PPI datasets. This integration aimed to identify DNV restriction factors that undergo regulation throughout the infection process and modulate their responses via physical interactions with DNV proteins. The analyses unveiled a total of 264 host factors that exhibit inhibitory effects on DNV. Among these, 59 displayed alterations in expression or abundance throughout the infection, and 41 were found to engage in physical interactions with DNV proteins. The genome-wide screening approach allowed for the identification of not only innate immune regulators and effectors, such as STAT2 or IFITM3, but also non-immune factors and networks. These include mitochondrial and cholesterol homeostasis, which were not previously associated with DNV restriction. These meta-analyses offer a systematic understanding of the inadequately characterized mechanisms employed by the host to control DNV. They also bring attention to potential targets that could inform the design of virus- and host-directed antiviral therapies.

## 5. Discussion

There can be little doubt that during the pandemic a multidisciplinary approach was taken by multiple laboratories across the world. A huge amount of data were produced, analysed, and applied/translated at extraordinary speed. Novel technologies and approaches were used to great effect and, as demonstrated here, creatively applied to existing and emerging challenges, often though successful collaborations. To keep this momentum going, the scientific community needs to continue these collaborative, multidisciplinary approaches; embrace novel methodologies and technologies with a view to applying them to a range of new and existing challenges; and, overall, be willing to engage with the public and policy makers to enhance the translation from basic scientific research to impacts on patient care and public health.

To be prepared for the next pandemic, it is essential for scientists to take forward the lessons learnt and think of applying multiple technologies and approaches to solving challenges in the future. Behavioural practices, such as isolating the sick and wearing face masks, have been found to be effective throughout history and provided an important buffer in the wake of the SARS-CoV-2 pandemic. The application of existing technologies such as ChAdOx and mRNA vaccines, lateral flow antigen detection, and information-sharing platforms were utilised to full effect. This was due to a colossal effort of the scientific community to focus on working together through the rapid sharing of resources, information, and sero-surveillance findings, as well as being open to discussions with the public and politicians. Moving forward, these open practices and willingness to try different applications of existing and developing technologies, as well as delving into the enormous body of data collected during the pandemic by the scientific community, can effectively communicate to the policy makers, funders, and other stake holders the best avenues to ensure a rapid response.
